# Bilateral Diffuse Leukoencephalopathy in an Electrocuted Patient Where the Brain Was Not in the Pathway of Conduction

**DOI:** 10.7759/cureus.33771

**Published:** 2023-01-14

**Authors:** Jamie Jacobs, Kyla Price, Minette Lagman, Jain Shreya, Antonio K Liu

**Affiliations:** 1 Internal Medicine, Adventist Health White Memorial, Los Angeles, USA; 2 Family Medicine, Adventist Health White Memorial, Los Angeles, USA; 3 Family Medicine, AltaMed Los Angeles, Los Angeles, USA; 4 Neurology, Adventist Health White Memorial, Los Angeles, USA; 5 Neurology, Loma Linda University School of Medicine, Loma Linda, USA

**Keywords:** diffuse leukoencephalopathy, neuron, leukoencephalopathy, cerebral leukodystrophy, mri, electrocution

## Abstract

Electrocution, damage caused by electric current passing through the body, is usually a serious event causing significant morbidity or even mortality. Graded damage is seldom encountered. According to Ohm’s law, the current is directly proportional to the applied voltage and inversely proportional to the resistance of a circuit. Electric current is expected to travel through cells that have the least resistance. Therefore, cells that allow action potential to travel down their cell membrane are presumably the ones with the least resistance. Among these are neurons and cells within the cardiac conduction system. Within a neuron, the axon will conduct electricity better than the cell body. While there have been a few cerebral white matter lesions caused by electrocution described in the literature, the mechanism is not fully understood. We report a patient with bilateral symmetrical subcortical abnormality where the electric current entered one hand and exited through her legs without affecting the head directly. We reviewed the literature and we hope it will further our understanding of how electrocution affects the central nervous system and which groups and parts of neurons are more susceptible than others.

## Introduction

Electrical shocks and lightning strikes pose serious and potentially life-threatening consequences to the human body. The consequences of exposure to these forms of electrical energy include cardiac and respiratory paralysis, thermal burns, neuropathy, and neurologic complications that can vary drastically from person to person [1,2]. Specifically, in regard to lightning strikes, these neurologic complications have been characterized into four categories. These four categories include immediate and transient symptoms, immediate and permanent complications, delayed syndromes, and destructive injuries secondary to falls or being thrown to the ground [3]. Factors that have been linked to the severity of the consequences include the magnitude of the energy delivered, resistance to current flow, type of current, duration of contact, and current pathway [1,2,4].

A previous case report documents a unilateral hemispheric leukoencephalopathy in a nine-year-old female with a reported electrocution at eight months of age. The parents reported that she was lying on her side next to a window when the lightning struck. The presumed point of entry was her right head. Years later, she was being worked up for headaches, which led to the incidental MRI finding of right hemispheric only diffuse white matter changes [5].

We present the first reported case of global, diffuse, and symmetrical leukoencephalopathy as a result of an electrical accident. There are three interesting features. First, her head was not involved. Second, the lesions are bilateral and symmetrical. Third, not all white matter areas or long tracks are affected to the same degree, showing a difference in susceptibility.

## Case presentation

**Figure 1:**
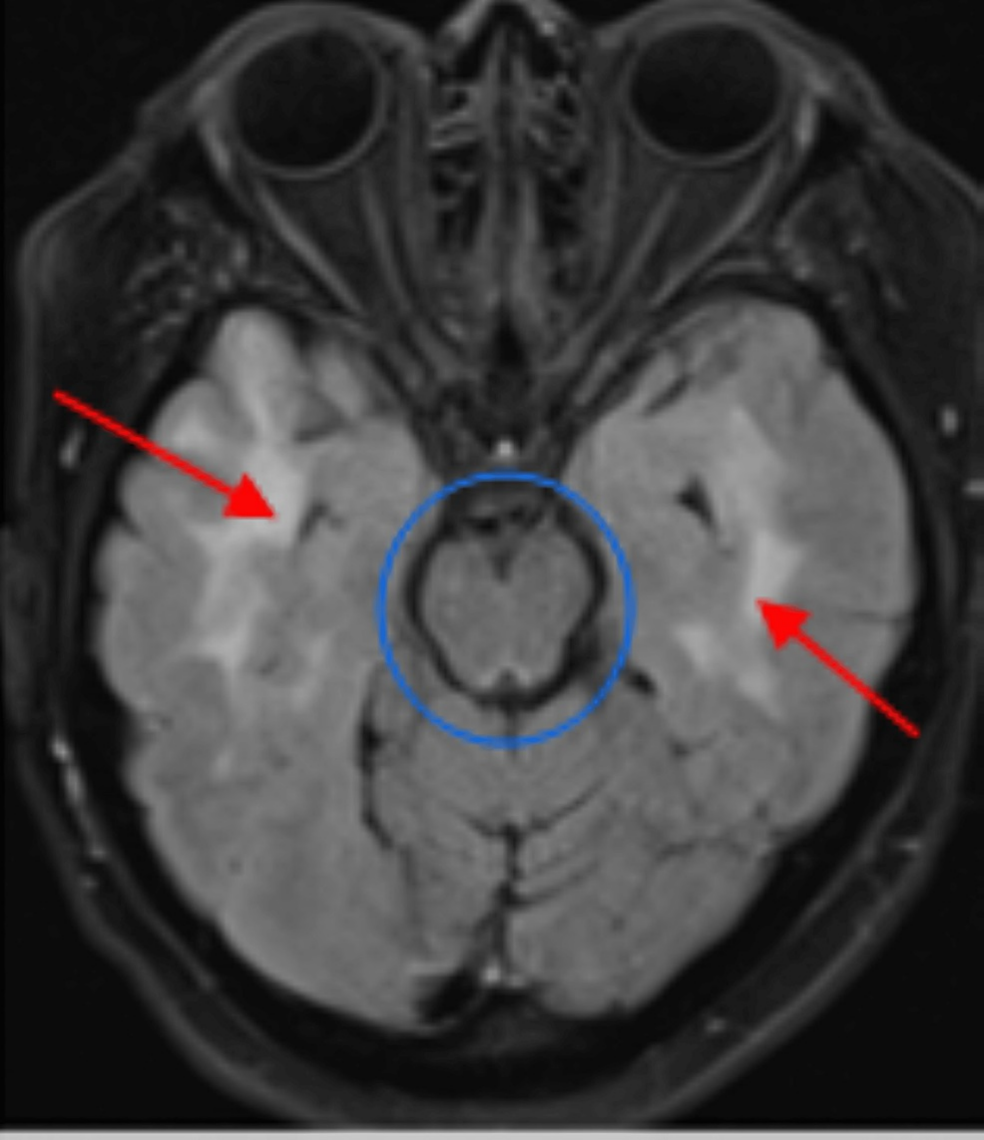
MRI FLAIR sequence showing white matter signal abnormality (red arrows) sparing midbrain (blue circle). FLAIR: fluid-attenuated inversion recovery.

**Figure 2:**
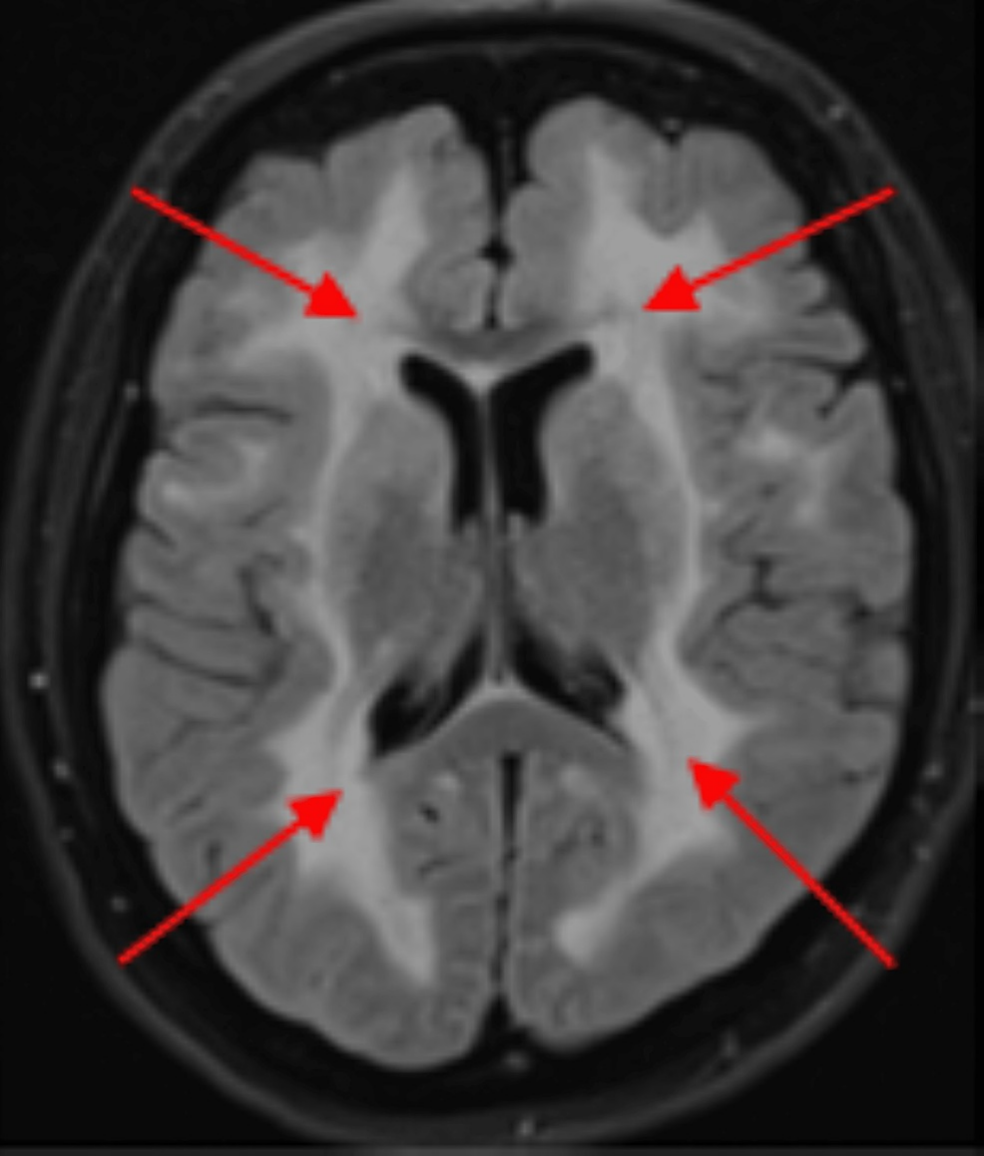
MRI FLAIR sequence showing extensive signal abnormality in the white matter (red arrows). FLAIR: fluid-attenuated inversion recovery.

**Figure 3:**
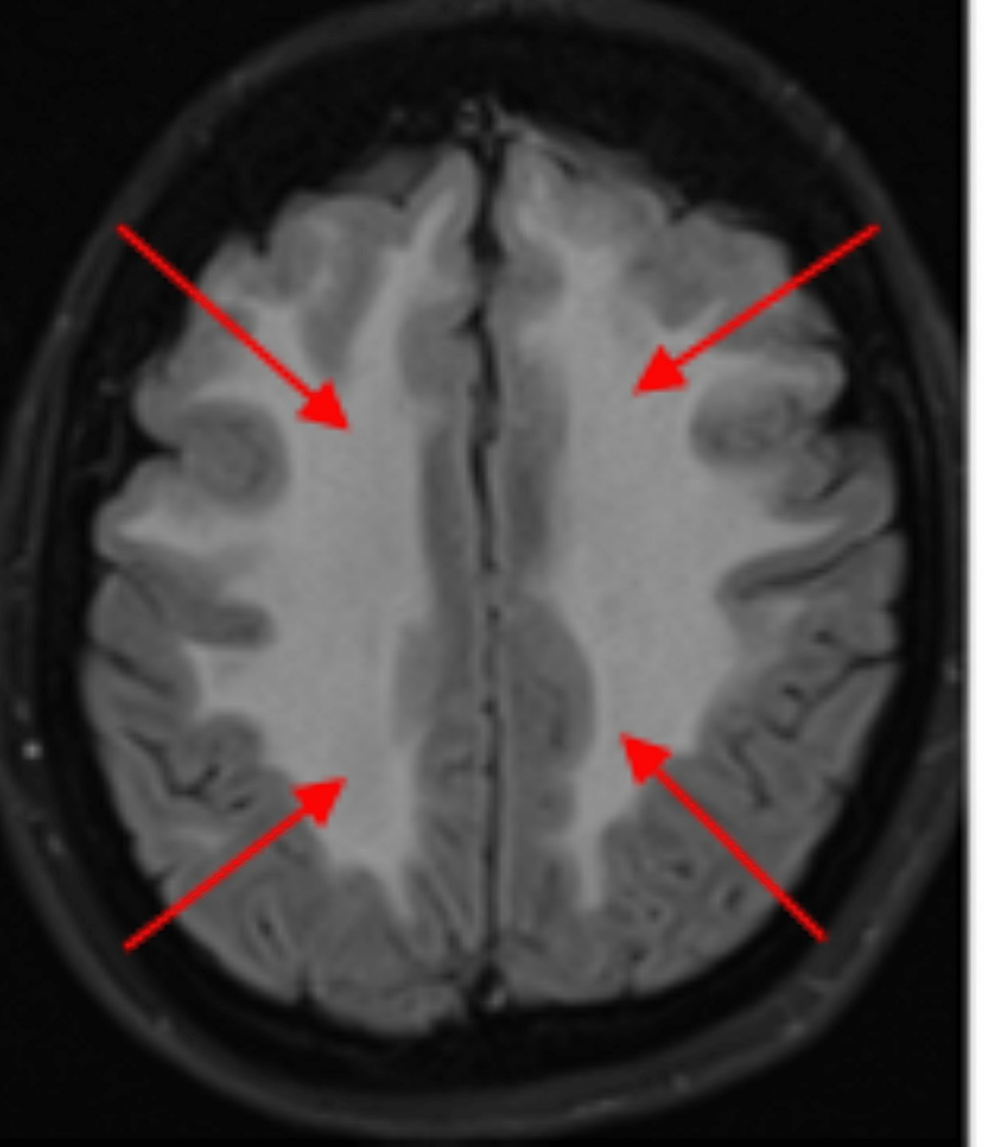
MRI FLAIR sequence showing extensive white matter signal abnormality (red arrows). FLAIR: fluid-attenuated inversion recovery.

The patient was a 53-year-old female who presented with new-onset vertigo and non-specific numbness. There was no significant past medical history. Her general examination was unremarkable. She had normal vital signs, normal mentation, and orientation. Her neurological examination was non-focal and non-revealing. Her dizziness and other subjective complaints were resolved shortly after admission. During her hospitalization, a brain MRI incidentally revealed diffuse bilateral symmetrical leukoencephalopathy with sparing of the cerebral cortex, brain stem white matter, cerebellum, internal capsule, thalamus, basal ganglia, caudate nucleus, and corpus callosum on fluid-attenuated inversion recovery (FLAIR) sequence (Figures 1-3). All other sequences, including diffusion-weighted imaging (DWI), apparent diffusion coefficient (ADC), susceptibility-weighted imaging (SWI), and T1, were unremarkable. Her laboratory workup, including an HIV test, was negative. Upon further questioning, the patient reported an electrocution injury at the age of eight years during which she touched a live antenna. She was shocked and had a loss of consciousness requiring hospitalization. She recovered well and reported no deficits due to the electrocution. She finished high school and had a normal, independent adult life, raising two children. This MRI was her first brain scan of any kind.

## Discussion

The term leukoencephalopathy refers to all brain white matter diseases and has a very broad differential including hereditary and acquired conditions [6,7]. The time of the patient's electrocution accident predates the availability of MRI. Therefore, it is impossible to determine definitively if the extensive white matter changes seen in the patient were a pre-existing condition or not. However, the patient denied cognitive or motor dysfunction in childhood; she also became a normal functional adult contributing to society. This makes a pre-existing hereditary leukoencephalopathy or leukodystrophy unlikely. In regard to acquired conditions, autoimmune, cardiovascular, infectious, and toxic metabolic causes were considered. However, white matter changes with these etiologies (such as multiple sclerosis or post-event demyelination) have a heterogenous "patchy" appearance on MRI. This patient's leukoencephalopathy had a homogenous appearance that also spared the brainstem. The patient was neither immunocompromised nor was she hypertensive. With a negative neurological examination and further blood work being nonrevealing, most entities on the differential diagnosis have been ruled out.

In our case, our patient was electrocuted when she was eight years old and her brain was not in the primary pathway of the electric current traveled. For lighting strikes, damage to one side of the brain can be explained by the fact that one side of the brain is in the pathway of conduction [5]. In our patient, it is demonstrated that the brain can still be damaged when it is not in the pathway of conduction. After the electricity in our patient reached the spinal cord from her finger, most of it probably went down to the ground but some parts continued to travel back up to the brain. Sensory nerves will naturally take the signal up to the brain, but motor nerves and all other axons are capable of conducting electricity “unnaturally” back up to the brain too. During the nerve conduction test, we know electrical impulses travel distally and antidromically away from the point of stimulation [8]. That explains why the brain was involved despite it not being the point of entry for electricity. Furthermore, since all the axons are recruited at the spinal cord levels, electricity will travel up to both sides of the brain, hence bilateral and symmetrical involvement. Our case may be one of the few, if not the only one, that proves radiologically such a phenomenon occurred.

The pattern of damage is predominantly subcortical in nature, i.e., leukoencephalopathy. However, was this a demyelination or leukodystrophy? The age at which different parts of the brain myelinate is a complicated subject. We have some knowledge of when myelination starts in certain parts of the nervous system [9]. However, there are few articles that describe when myelination is complete. It is known that adults with electrocution can have transverse myelitis [10]. Our patient was electrocuted at the age of eight years. Myelination had certainly begun but was not completed. Was it a destructive process such as demyelination or abnormal myelin formation - leukodystrophy? That will remain to be determined. Whatever it was, it did not prevent our patient from achieving further developmental milestones or attaining normal independent adulthood.

Not all white matter areas are affected equally. Electrocution is often either fatal or too minor to leave any injury signature. Rarely, there are survivors that show graded damage. In our patient, while the subcortical area appears to be uniformly affected, there is a sparing of the internal capsule, corpus callosum, and most of the long tracks in the midbrain, pons, and subcortical cerebellum. This pattern appears to generate more questions than answers. Is the degree of myelination of different neurons the major factor that determines susceptibility? Or, is the determining factor the degree of connectivity to different parts of the brain?

## Conclusions

We presented a patient who was incidentally found to have impressive white matter signal abnormality, presumably from early-life electrocution. The brain was not in the pathway of electricity. This case demonstrates that electrocution damage can happen even if the brain is not in the expected pathway of conduction. We hope that the bilateral symmetrical distribution as well as different regional susceptibility will shed light on the effect of electrocution on brain myelination.
